# Supplementary material for: Simulating emergence of novelties using agent-based models
Source: PLoS One. 2023 Dec 11;18(12):e0294228. doi: 10.1371/journal.pone.0294228 (PMC10712848; doi:10.1371/journal.pone.0294228)
Supplement: Attachment — Submitted filename: Response to Reviewers.pdf [file pone.0294228.s001.pdf]

Response to Reviewers

We would like to thank the reviewers for kindly reading our manuscript and providing detailed and helpful feedback. We have taken careful consideration of all of the points raised and have addressed them in detail.

To highlight some of the main changes to the manuscript: we have better defined our work with respect to the existing literature by including an additional model in our comparisons, we have provided a more detailed explanation of the mechanisms behind the proposed model, and we have updated the future work and limitations section to address the limitations of the model and our analysis (namely, mind reading and how the results are dependent on the choice of strategy).

Changes to the manuscript have been formatted in the following way: changes suggested by the reviewers are marked in blue, with each change corresponding to a specific comment. In addition minor changes (such as correcting grammatical errors, making explanations more clear, etc.) have been marked in red.

Below, we address each of the reviewers' comments.

Reviewer 1

[R1-Ma1]: In a Polya's urn model the content of the urn is hidden. What we measure is the stream observed as the result of picking up elements from the urn. All the quantities related to innovation, Heaps', Zipf's and Taylor's laws are determined from the stream not from the urn. If there is only one urn I do not see any conceptual problem. When we have different urns associated to different agents, we should be careful to avoid mind reading. We are not allowed to look into other's head, the urn in this case. In iteration one, for example, agent A adds \nu+1 new agents that are in the urn of B and that were never observed. Agents E and F are in B's head and not accessible. They are in the adjacent possible of B, so that even B has never observed them. How do you avoid mind reading? Can you modify the model to solve this problem?

Reply: We thank the reviewer for bringing up the interesting and important point of mind reading in these models. This problem can be defined as the following: consider three agents, A, B and C. In the models discussed in this work, A is able to recommend B to C, even if A has never interacted with B. This recommendation is not logical.

As this concern affects the two existing models and the proposed model, we have left it to future work to understand the effect of mind reading in these models. To address this issue, a paragraph has been added to the Limitations and Future Work section, explaining this problem and how it could be overcome. Namely, the memory buffer exchange should be limited to agents in the agent's actual space, and not in the adjacent possible space.

Reviewer 2

[R2-Ma1]: Lines 55-58. Here you mention your previous work [20], in which you already change the rule to choose the caller. In that previous paper you have already found a model which has "created asymmetries in the semantics, thereby leading to waves of novelties". The results you obtained there leveraged on the same type of analysis. In this new paper, you are again proposing a modification of the caller mechanism to reproduce waves of novelties, this time introducing labels. In order for this paper to be original and substantial new work, you need to better describe what you have done in the other paper, and what maybe has failed there and you are instead recovering in this paper. Otherwise it seems just an alternative solution to the same problem that has already been solved in another paper, and as such it should be stated somewhere. With the same argument, I would like to see a comparison between these two modifications of the model, so that the reader understands what is the improvement brought by the model proposed here with respect to not only the original model by Ubaldi et al, but also the generalized ABM proposed in the cited paper. This is even more important considering how, in the discussion, you mention that including biased strategies like the one in the cited paper can improve the results. If the authors decide not to compare these models, which I recommend against, they should sufficiently explain why and provideat least a sufficient analysis of the difference between the two models.

Reply: We thank the reviewer for raising this important point. In the original manuscript we failed to properly explain the previous model that was also introduced by us. To remedy this, we have added a detailed description of that model, referred to as the *Suda et al. model* and have included it in all of the subsequent analysis. We have described how the Suda et al. model failed to capture waves of novelty and why this work was necessary to capture waves of novelty and more accurately capture the behaviour of the real data. This allowed for a more fair comparison and highlights the improvements introduced by the proposed model. We hope that with this change, the originality of this work is more clear.

[R2-Ma2]: Proposed model description and figure1 (Lines 156-191). There are some things that are not clear to me.

- First of all, in your model, it seems like N_L is always nu+1, since this is the number of new agents triggered by calling an agent for the first time and to which you assign the same label. Is so, it should be stated.
- Then, by looking at the example of the figure, when you count the number of agents not with label L, you are considering only agents that have been called at least once before. For example, in iteration 3, you are not counting C, D, F, G, H. So basically this counts the total number of activated agents in the system, minus the nu+1 agents sharing the same label L. It this correct? You might want to clarify all these points in the text.
- Moreover, it is not clear to me why you choose such functions f and g, and why you choose those weights in the various classes. Can you add an intuitive explanation of these functions? For example, for nodes in class 3 you assign weight eta*f, i.e., \frac{\eta N_L}{N_L + \eta (N-N_L)}. Can you explain the role of \eta and of f in this weight function?

Point is that these new weights might work perfectly looking at the results, but if they have no connection to the phenomena you want to describe, then the model has a low value.
- What is the "physical" mechanism controlling the weights you have introduced? What happens, for example, when eta is 0 or 1? Same for zeta and the other weights. More in general, you should a deeper explanation of why you divide the possible new callers into these classes that depend on the label of the last caller.
- In line 183 you talk about the two parameters effect on the propensity to exploit vs explore, but it is not clear how they do so, based on the model definition. This is later explained in the results, but it is important to provide a first interpretation of the possible outcomes based on the model definition. Can you state if you tend to explore more with higher or lower values of each parameter, giving some examples of how the weights would be modified?

Reply: We thank the reviewer for raising multiple important points here.

It is correct that N_L wil always be nu+1. We have rephrased the paragraph to include this explicitly. It is also correct that N_L bar is the total number of agents in the system minus N_L. We have updated the paragraph to also include this explicitly.

Regarding the choice of f and g, the functional form of f and g come from the work of Monechi et al [19], who derive that with this choice of f and g, the growth of the number of distinct elements with time follows Heaps law, as required by models which aim to reproduce the dynamics of social networks. To add an intuitive explanation of these functions and the choice of parameters zeta and eta, we have added a paragraph which describes the behaviour of f and g for various values of the parameters zeta and eta. For example, we explain how the likelihood of selecting a past caller increases and zeta as (zeta, eta) increases from (0,0) to (1,0). In addition, we have included a table which shows the selection probabilities of the five classes for the limits of zeta and eta.

A deeper explanation has also been given as to why we split the agents into five classes.

[R2-Mi1]: Line 12-15 Original Polya's urn with two colors (black and white) does not reproduce these laws, which foresee a power-law behavior. This is instead obtained when considering a very large number of initial colors or when considering generalization of the Polya's urn, like the ones you later cite. In my opinion, citations 14-18 are not so appropriate to what you have stated in this paragraph, unless you meant to say something else that I have misinterpeted. For example, the Matthew effect you cite does not talk about Zipf or Heaps, nor Polya's urn.

Reply: We thank the reviewer for raising this point. We have removed the sentence about Polya’s urn model reproducing Zipfs and Heaps law. We have also removed the inappropriate citations.

[R2-Mi2]: Line 36 "Kaufman" typo, missing f.

Reply: The typo has been corrected.

[R2-Mi3]: Line 38-39 Paragraph is not clear, and I don't understand what "respectively" refers to here.

Reply: We thank the reviewer for pointing this out. We have made the paragraph more clear, and removed the use “respectively”

[R2-Mi4]: Line 40-43 You are repeating what said in lines 38-39, but using less precise terminology. It is not clear what an agent is in the context of the system/urn, since you had not talked about an agent before. In fact, the growing system can be anything, could be for example the knowledge of a person or the number of inventions of a population, with balls representing keywords, songs, patents, etc. You need to clearly state here that in this work you are going to study a social system, and that for you the urn represents the space of possible contacts of an individual, and each color is a person. As a matter of fact, you make the assumption ball=agent throughout the paper. This specific interpretation is key in Ubaldi et al, but not in the original paper by Tria et al. You might want to say first that Ubaldi et al. extended the urn model with triggering to reproduce the growth of a social system, considering different urns, representing the space of possibilities of different agents, with the color of balls representing various agents. And then you can further notice that the ball itself does not represent the agent. Rather, the color or ID of the ball represents the agent. In fact, there can be many balls with the same color/ID representing the same agent. Since all this is the base of the results, it should be as clear as possible for the reader.

Reply: We thank the reviewer for this important point.It is important for the reader to understand that in this context the agents refer to individuals in a social system. We have rewritten this paragraph to highlight that the Ubaldi et al. model was used to model social systems and that the model proposed aims to better capture the behaviour of real social systems. We have rephrased the paragraph to highlight the fact the caller and callee pair represent individuals. As suggested by the reviewer, we have updated the manuscript to mention how it is the ID of the ball that represents the agent and not necessarily the ball.

In addition lines 40-43 have been rewritten to be more clear and reduce unnecessary repetition.

[R2-Mi5]: Line 120-124. The nu+1 new balls are added only if the pair (caller, callee) is activated for the first time. All subsequent times, they only reinforce with rho balls of the other. I hope you have not done this mistake also while coding, but I cannot check since no code has been provided. Please share the code for open reproducibility of your results.

Reply: We thank the reviewer for highlighting this error. It is correct that the nu + 1 balls in the memory buffer are only exchanged when the interaction happens for the first time. This was just a mistake in the paragraph and has been corrected. We have also confirmed that this error is not present in the code. In addition, the code has been made publicly available, with an additional subsection added to the methods section highlighting this. The code can be found here: https://github.com/tsukuba-websci/UrnBasedDynamicNetworkModel

[R2-Mi6]: Figure 1 typos: top left panel "an possible relation". Repeatedly said parter instead of partner. Top right there is a smaller table class1 to weights table to be removed. Iteration 2 in the network E should be colored. Iteration 3 the numbers in f and g are wrong (0s should be 1)

Reply: We thank the reviewer for pointing out the mistakes in figure 1. We have corrected the grammatical error to “a possible relation”. The word parter has been corrected to partner. We have coloured the E ball in iteration 2 and removed the small class weights table. We have corrected the values in f and g in interaction 3.

[R2-Mi7]: Line 212 Cluster coefficient is more commonly referred to as clustering coefficient.

Reply: We have rephrased cluster coefficient to clustering coefficient.

[R2-Mi8]: Line 259: maybe you wanted to say interval I_i?

Reply: We thank the reviewer for highlighting this typo. We have replaced I with I_i.

[R2-Mi9]: Line 259-260: the way it is phrased, you are considering the node with the highest number of interactions in the whole growth time step. I suppose you instead meant the highest number of interactions in the interval I_i, like in Monechi et al.

Reply: We thank the reviewer for highlighting this. The recentness metric that we use in this work is the same as that of Monechi et al. We have rephrased our description of the recentness metric so that this is more apparent and its description is more clear.

[R2-Mi10]: Line 262. Forgotten to add the paragraph label Local entropy?

Reply: We have added the paragraph label for local entropy.

[R2-Mi11]: Line 266-267: maybe you wanted to say "node j"?

Reply: We thank the reviewer for noticing this mistake. We have changed node I to node j.

[R2-Mi12]: Line 282 What are node species?

Reply: We thank the reviewer for pointing this out. It’s not clear what species means. In this context, we mean the distinct number of nodes. We have removed the word species and have replaced it with *total number of distinct nodes*.

[R2-Mi13]: Line 293. In this distance metric, you should say how each property is normalized. If the measures do not take values in the whole spectrum [0,1], then it should be clearly stated. For example, in the definition of OC, OO, NC and NO in line 228-229, you say that these are the frequencies, not necessarily normalized, so could be huge values.

Reply: We thank the reviewer for highlighting the issue in our description of the measure. The metrics NO, NC, OO, OC are normalized by the total number of interactions, so that they take values between 0 and 1. We have made this more clear in the description of these 4 metrics. We have also added a line to the description of the measure to emphasize that all of the metrics in the measure take values between 0 and 1.

[R2-Mi14]: Line 295-297: also include the other two parameters you are adding in the proposed model

Reply: The other two model parameters, zeta and eta, were added.
[R2-Mi15]: Line 313-320 and Table 1. The parameters shown in Table 1, in particular nu, are outside the range you state in these lines. So which parameters are you using for the original model? If the maximum value considered is nu = 20, you should actually increase it further, because you are finding a best fit at the edge of the space of the parameters, showing that the best fit could be outside the set of parameters you have tested.

Reply: We thank the reviewer for noticing the typo in the experiment parameter limits.

In addition, the reviewer suggests that because the optimal parameters were found to be on the edge of the parameter space, we should increase the upper limits of the parameters. As suggested, we have increased the limits of rho and nu from 20 to 30. We have found again that the value of rho tends to the upper limit of the parameter space. However, it is worth pointing out that nu also changes accordingly, keeping the ratio of rho/nu roughly constant. For instance, when the upper limits were set to 20, the optimal parameters for the Twitter Mentons Network were rho/nu = 10/19 ~ 0.53. Similarly, when the upper limits were set to 30, the optimal parameters of the Twitter Mentions Network were set to rho/nu = 18/30 = 0.6. For this reason, we find it sufficient in this work to keep the upper limits of the parameters rho and nu to 30.

[R2-Mi16]: Line 318-320: As for the strategy s, why have you chosen the strategy SSW? You have stated elsewhere that the strategy chosen can influence quite a lot the way the network grows and I agree. As reported in the original paper by Ubaldi et al, they found the best strategy to be ASW for the APS subsample and WSW for the Twitter Mentions Network. Have you tried other strategies? Does the choice of the strategy change the results? If so, how? Mention this in the limitations of the study or provide further evidence.

Reply: We thank the reviewer for highlighting that the choice of strategy will affect the results and hence we have to better justify our choice of strategy. In response to this we have decided to choose the optimal strategies from the Ubaldi et al. model (s=ASW for the APS subsample and s=WSW for the Twitter Mentions Network) and from the Suda et. al. model (s_c = PGBK). The choice of parameters are now summarised in table 1. In addition, we have added a paragraph to the Limitations and Future Work section to highlight how our analysis used only a single strategy s for each datasource, and that it would be interesting in future work to consider how the results would be affected by other strategies.

[R2-Mi17]: Line 354 typo in zeta

Reply: The formatting of zeta has been corrected.

[R2-Mi18]: Figure 4 and 5. They seem to be interchanged, since in the text you refer to Fig 4, but it is Fig5.tif

Reply: We have corrected the figure numbers.

[R2-Mi19]: Lines 378-390 and related figure. This figure is very hard to read and could be misleading. For example, I could say that also for the proposed model there is some

skewedness, where higher active frequencies are found only in low birth times, which are not seen in the empirical distribution. You should state this issue. Moreover, the panels have different scales and limits in the y axis. This does not help in the interpretation. You could add that in the existing model there are some points with very high frequency (higher than the highest in the empirical), and are all among the first birth steps. After 100 steps, the distribution seems very similar to the proposed model. Moreover, the scatter points are all the same, and it is hard to understand the density of points. You could use a color scale for the points, depending on the density of points, add an alpha, or consider some binning or cumulated distributions. This could help visualize better the data and interpret it.

Reply: We thank the reviewer for their comments regarding the figure. The paragraph has been rephrased so that it better represents the figure. We have also addressed the limitation regarding the models all having highly active early occurring agents.

The graphs have been changed so that they now have the same limits and scales. In addition, we have added opacity to the points to help showcase the density of points in the figure.

[R2-Mi20]: Line 396-397 How do you define the maximum and minimum values among all the lines? For example in the related figure it seems there are other gray lines above the red line or below the blue one. Also, to which combination of nu and rho they refer to?

Reply: We thank the reviewer for mentioning the issues with the graphs. The definition of the minimum and maximum was unclear and so it has been updated. The red line indicates the pair rho, nu which takes the absolute maximum value at any point zeta/eta. Similar for the minimum. We have also updated the figure to include which values of rho and nu which correspond to these maximum and minimum lines.

[R2-Mi21]: Figure 7 and 8. Better to delete the "(log scale)" from the ylabel, because otherwise one can interpret the numbers in the y axis to be considered in logarithm, i.e., you are plotting the logarithm of the class size. So for example in class 1 at 20k, it could be interpreted as log(class_size) = 100k. Furthermore, for better clarity, you should do the zoom for the cluster size as you did in the other figure. This should help to see how class 2 + class 4 is always nu+1

Reply: We thank the reviewer for pointing out the issues with figure 7 and 8. We have removed the words “log scale” from these figures. In addition, we have provided a zoom in fig7 class size so that it is more clear that class 2 and class 4 take a maximum value of nu + 1. The caption for the figure has been updated to reflect the addition of the zoomed in graph.

[R2-Mi22]: Line 412-422 Here are you referring to a specific model simulation? With which parameters?

Reply: We thank the reviewer for highlighting that we did not specify the model/ parameter set for this analysis. In the discussion section, we analyse the ability of the proposed model to generate waves of novelty. In this analysis, we used the proposed model with the optimal parameter set as shown in table 5 for both Twitter and the APS Co-authors Network. We have updated the paragraph to include this information.

[R2-Mi23]: Line 427 missed closed parenthesis

Reply: We thank the reviewer for highlighting this error. The closing parentheses has been added.

[R2-Mi24]: Line 468-470 Add that a circle represents a time step in which the selected agent is not active.

Reply: We thank the reviewer for highlighting this error. We have included a circle to represent the period where the agent is inactive.

[R2-Mi25]: Figure 9 caption. There are some mistakes and incomplete. The fact that it has a sudden increase does not indicate that it is classified as class 2, rather that as soon as it called, it is has a burst in activity as a caller (blue triangles up). After this it remains in class 3 and is only called some other few times. Also, you should mention and explain what you are plotting in panel b in the caption.

Reply: We thank the reviewer for highlighting that the caption for fig9 was incomplete. We have provided a more detailed explanation of what the figure is showing, including a more detailed explanation of the burst of activity the agent receives. A description of (b) is also provided in the updated caption. In particular, the red point in (b) is the agent whose history is plotted in (a). This shows that the agent was the most active agent in that time interval and can therefore be considered as triggering a wave of novelty. In addition, we have changed the layout of the figure so that (b) is now below (a). In this was it is more clear that this agent's burst of activity corresponds to it being the most active agent in that time period.
